# Cytokines and Water Distribution in Anorexia Nervosa

**DOI:** 10.1155/2021/8811051

**Published:** 2021-04-02

**Authors:** Hubertus Himmerich, Bethan Dalton, Olivia Patsalos, Ulrike Schmidt, Iain C. Campbell

**Affiliations:** ^1^Section of Eating Disorders, Department of Psychological Medicine, Institute of Psychiatry, Psychology & Neuroscience, King's College London, London SE5 8AF, UK; ^2^South London and Maudsley NHS Foundation Trust, London SE5 8AZ, UK

## Abstract

In patients with anorexia nervosa (AN), decreased intracellular (ICW), extracellular (ECW), and total body water (TBW) as well as changes in serum cytokine concentrations have been reported. In this exploratory study, we measured body composition and serum cytokine levels in patients with AN (*n* = 27) and healthy controls (HCs; *n* = 13). Eating disorder symptom severity was assessed using the Eating Disorder Examination-Questionnaire (EDE-Q). Body composition was determined by bioimpedance analysis (BIA) which provided information on ICW, ECW, and TBW. Following blood collection, 27 cytokines and chemokines were quantified using multiplex ELISA-based technology: Eotaxin, Eotaxin-3, granulocyte-macrophage colony-stimulating factor (GM-CSF), interferon- (IFN-) *γ*, interleukin- (IL-) 1*α*, IL-1*β*, IL-2, IL-4, IL-5, IL-6, IL-7, IL-8, IL-10, IL-12/IL-23p40, IL-12p70, IL-13, IL-15, IL-16, IL-17A, interferon *γ*-induced protein- (IP-) 10, macrophage inflammatory protein- (MIP-) 1*α*, MIP-1*β*, monocyte chemoattractant protein- (MCP-) 1, MCP-4, thymus and activation-regulated chemokine (TARC), TNF-*α*, and TNF-*β*. ICW, ECW, and TBW volumes were significantly lower in patients with AN than in HCs. In the whole sample, GM-CSF, MCP-4, and IL-4 were positively, whereas IFN-*γ*, IL-6, and IL-10 were negatively associated with all three parameters of body water. In AN participants, we found a statistically significant negative correlation of IL-10 with ICW, ECW, and TBW. Our results suggest an interaction between body water and the cytokine system. Underlying mechanisms are unclear but may involve a loss of water from the gut, kidneys, or skin due to AN-associated inflammatory processes.

## 1. Introduction

Anorexia nervosa (AN) is characterised by significantly low body weight, an intense fear of weight gain, and disturbed body perception [1]. Compared to healthy individuals, the overall mortality in people with AN is significantly increased with a standardized mortality ratio of ~5 [2]. AN is often accompanied by significant physical health problems, including renal insufficiency, urolithiasis, and disturbances in water and electrolyte balance, e.g., dehydration [3–6]. Bioimpedance analysis (BIA) allows assessment of water distribution and balance in the body, i.e., intracellular (ICW) and extracellular (ECW) water and total body water (TBW). In previous studies, decreased ICW, ECW, and TBW were found in patients with AN [7, 8], and these have been reported to increase during therapy and refeeding [9].

A second important etiopathological factor contributing to AN is inflammation. Meta-analyses indicate that AN is associated with elevated concentrations of the proinflammatory cytokines tumor necrosis factor- (TNF-) *α* and interleukin- (IL-) 6 [10, 11]. Such inflammatory molecules can be produced by macrophages in the periphery and by astrocytes and microglia in the brain and have been reported to affect systems with a role in the development of AN, i.e., by regulating appetite and food intake, mood, and cognition [12–17].

Although inflammation involving excessive production of proinflammatory cytokines and disturbances in the fluid balance have both been reported [7–11], a potential association between cytokine release and water balance has not been explored in patients with AN. The ratio of ECW/TBW, i.e., the oedema index, has been reported to indicate the severity of malnourishment in severely sick patients, independent of their diagnosis, and to be associated with low serum albumin and haemoglobin [18]. Peripheral or pulmonary oedema can be a serious problem in AN, specifically during refeeding [19]. In AN, oedema may reflect heart problems [19] or a deficiency of osmotically potent molecules such as albumin and haemoglobin [6].

Therefore, we analysed body composition data related to water balance (ICW, ECW, TBW, and the oedema index) and potential associations of these parameters with cytokine concentrations in patients with AN.

## 2. Methods

### 2.1. Participants

Participants with AN (*n* = 55) and healthy controls (*n* = 30) were recruited as part of a larger study (for full study details, see [20, 21]). Female adults with a primary diagnosis of AN and a BMI < 17.5 kg/m^2^ were recruited from Specialist Eating Disorder Services in and around London. HCs, free from a history of or current mental health disorder and physical illness, were recruited via an e-mail circular to students and staff at King's College London. Informed consent was obtained from all participants. The study was conducted in accordance with the Declaration of Helsinki, and the study received ethical approval from the South East London Research Ethics Committee (REC ref: 09/H0807/4).

To be eligible for the present analyses, AN participants were required to be free from autoimmune and/or inflammatory diseases and HCs were required to have a BMI within the healthy range (BMI 18.5-24.9 kg/m^2^), leading to the exclusion of five AN participants and one HC. Participants also had to have serum samples available for analysis, resulting in a final sample of 27 participants with AN and 11 HCs.

### 2.2. Measurements

Height, weight, and body composition were obtained for all participants. Body composition was measured using a portable and noninvasive BIA InBody S10 machine (Biospace Co., Ltd.). This provides data on a range of parameters, including intracellular (ICW) and extracellular (ECW) water, total body water (TBW), and the oedema index (ECW/TBW). Eating disorder symptom severity was assessed using the Eating Disorder Examination-Questionnaire (EDE-Q) [22]. Several additional measures were collected and are reported elsewhere (see [20, 21]).

Following blood sample collection, serum was stored at -80°C prior to use. Serum was thawed at room temperature, and 40 inflammatory markers were quantified simultaneously using multiplex ELISA-based technology provided by the Meso Scale Discovery V-PLEX Human Biomarker 40-Plex Kit (Meso Scale Discovery, Maryland, USA). Plates were scanned on the Mesoscale Scale Discovery MESO Quickplex SQ 120 reader at the Social, Genetic and Developmental Psychiatry (SGDP) Centre, Institute of Psychiatry, Psychology and Neuroscience, King's College London. As the focus of the current study was on cytokines, rather than the broader group of inflammatory markers measured, only data on the following cytokines and chemokines (*n* = 27) were used: Eotaxin, Eotaxin-3, granulocyte-macrophage colony-stimulating factor (GM-CSF), interferon- (IFN-) *γ*, IL-1*α*, IL-1*β*, IL-2, IL-4, IL-5, IL-6, IL-7, IL-8, IL-10, IL-12/IL-23p40, IL-12p70, IL-13, IL-15, IL-16, IL-17A, interferon *γ*-induced protein- (IP-) 10, macrophage inflammatory protein- (MIP-) 1*α*, MIP-1*β*, monocyte chemoattractant protein- (MCP-) 1, MCP-4, thymus and activation-regulated chemokine (TARC), TNF-*α*, and TNF-*β*. The cross-sectional comparisons of these data are reported elsewhere [23].

### 2.3. Statistical Analysis

Statistical analyses were performed in Stata 15 [24]. For demographic, anthropometric, and clinical characteristics, *t*-tests or Mann–Whitney *U* tests (depending on normality) were used to compare AN and HC participants. Due to the presence of outliers and nonnormal distributions, Mann–Whitney *U* tests were performed to compare cytokine data between AN and HC participants, and Spearman's rank correlations were performed to assess the relationship between body water parameters (ICW, ECW, TBW, and ECW/TBW) and cytokines (for the group as a whole and then for the patients and controls separately). The level of significance was set at *p* < 0.05, and as these are exploratory analyses, levels of significance were not adjusted for multiple testing.

## 3. Results

Demographic, anthropometric, and clinical characteristics are shown in Table 1. As expected, participants with AN reported significantly greater ED symptoms and had lower BMI and body fat percentages, compared to HCs. With regard to body water, ICW, ECW, and TBW were significantly lower in patients with AN than in HCs. There was no significant difference in the ratio between ECW and TBW.

Cytokine levels could not be detected in all serum samples. Therefore, Table 2 depicts the number of participants with undetectable cytokine concentrations for each cytokine in the whole sample. It also informs about the median serum concentrations of cytokines for the HC group and the group of people with AN. IL-6 and IL-15 levels were significantly higher in patients with AN compared to HCs, whereas TNF-*β* concentrations were lower in people with AN.

Tables 3 and 4 depict all correlations between cytokine concentrations and water distribution variables in the whole sample and in people with AN. In the following paragraphs, we will, however, give more detailed information on the significant correlations.

In the whole sample, ICW was significantly and positively correlated with IL-4 (*r*_s_(34) = 0.38, *p* = 0.0217), GM-CSF (*r*_s_(31) = 0.43, *p* = 0.0121), IL-1*α* (*r*_s_(36) = 0.33, *p* = 0.0409), TNF-*β* (*r*_s_(36) = 0.33, *p* = 0.0414), and MCP-4 (*r*_s_(36) = 0.33, *p* = 0.0448). ICW had a significant negative association with IFN-*γ* (*r*_s_(36) = −0.34, *p* = 0.0351), IL-10 (*r*_s_(36) = −0.45, *p* = 0.0047), IL-6 (*r*_s_(36) = −0.36, *p* = 0.0247), and IL-15 (*r*_s_(36) = −0.33, *p* = 0.0466). In AN participants, ICW was correlated negatively with IL-10 (*r*_s_(25) = −0.42, *p* = 0.0275), and in HCs, ICW correlated positively with GM-CSF (*r*_s_(9) = 0.66, *p* = 0.0260).

In the whole sample, ECW had a significant positive association with IL-4 (*r*_s_(34) = 0.43, *p* = 0.0083), GM-CSF (*r*_s_(31) = 0.45, *p* = 0.0093), and MCP-4 (*r*_s_(36) = 0.38, *p* = 0.0181). ECW significantly and negatively correlated with IFN-*γ* (*r*_s_(36) = −0.35, *p* = 0.0312), IL-10 (*r*_s_(36) = −0.45, *p* = 0.0047), and IL-6 (*r*_s_(36) = −0.34, *p* = 0.0354). In AN participants, ECW was associated negatively with IL-10 (*r*_s_(25) = −0.48, *p* = 0.0124), and positively with IL-4 (*r*_s_(23) = 0.44, *p* = 0.0288), and MCP-4 (*r*_s_(25) = 0.44, *p* = 0.0203). In HCs, ECW correlated with IL-4 (*r*_s_(9) = 0.61, *p* = 0.0454) and GM-CSF (*r*_s_(9) = 0.68, *p* = 0.0216).

**Figure 1:**
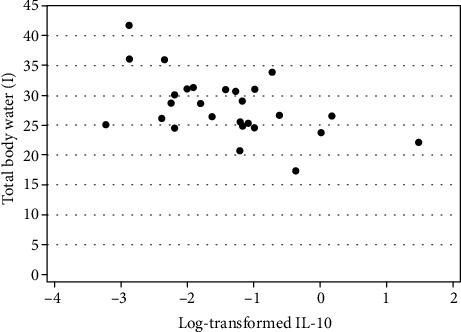
The association between total body water and IL-10 levels (log-transformed for improved presentation) in study participants with anorexia nervosa.

In the whole sample, TBW was significantly and positively associated with IL-4 (*r*_s_(34) = 0.40, *p* = 0.0168), GM-CSF (*r*_s_(31) = 0.43, *p* = 0.0138), IL-1*α* (*r*_s_(36) = 0.32, *p* = 0.0485), and MCP-4 (*r*_s_(36) = 0.37, *p* = 0.0241). TBW was significantly and negatively correlated with IFN-*γ* (*r*_s_(36) = −0.36, *p* = 0.0255), IL-10 (*r*_s_(36) = −0.46, *p* = 0.0039), and IL-6 (*r*_s_(36) = −0.37, *p* = 0.0215). In AN participants, TBW was negatively correlated with IL-10 (*r*_s_(25) = −0.44, *p* = 0.0215).  Figure 1 illustrates this negative correlation. In HCs, ICW correlated positively with GM-CSF (*r*_s_(9) = 0.66, *p* = 0.0260).

The ECW/TBW ratio was significantly and negatively associated with IL-12/IL-23p40 (*r*_s_(36) = −0.32, *p* = 0.0490), Eotaxin-3 (*r*_s_(36) = −0.34, *p* = 0.0372), and MIP-1*α* (*r*_s_(36) = −0.34, *p* = 0.0369). In AN participants, the ECW/TBW ratio was correlated negatively with MIP-1*α* (*r*_s_(25) = −0.43, *p* = 0.0241). In HCs, the ECW/TBW ratio was negatively associated with MCP-4 (*r*_s_(9) = −0.76, *p* = 0.0062).

As IL-10 was consistently associated with body water parameters both in the whole sample and the AN sample, we calculated correlations between IL-10 concentrations and body mass. In the whole sample, weight was correlated with IL-10 (*r*_s_(36) = −0.47, *p* = 0.0027). In AN participants, weight was significantly associated with IL-10 (*r*_s_(25) = −0.55, *p* = 0.0028).

## 4. Discussion

We replicated reports of decreased ICW, ECW, and TBW in patients with AN [7–9], i.e., the overall amount of water inside and outside of cells was decreased in patients with AN. This was not unexpected, as patients with AN have a lower total body mass and a lower total body volume. However, we found no differences in the oedema index between people with AN and HCs, i.e., there is no apparent shift in water from inside cells to the extracellular matrix.

In the whole study sample, several cytokines were significantly associated with ICW, ECW, and TBW. In particular, GM-CSF, MCP-4, and IL-4 were positively, whereas IFN-*γ*, IL-6, and IL-10 were negatively associated with all three body water measures. From studies in patients with inflammatory diseases, it appears that cytokines can promote water loss via the gut (by increasing intestinal permeability) [25], via electrolyte and water transport in the kidneys [26], via the lungs (by impeding the reabsorption of salt and water leading to an increased movement of fluid from the lung interstitium to the alveolar lumen [27] or via transdermal water loss [28]). Dehydration and changes in water distribution, in turn, may also influence inflammation and the production of proinflammatory cytokines. For example, water restriction is reported to lead to hypovolaemia and reduced kidney perfusion in AN, which can lead to tubular necrosis, repeated urinary tract infection, or nephrocalcinosis [29], and these conditions have been reported to be associated with changes in cytokine signalling [30–32]. However, these mechanisms of the mutual influence of cytokines and the fluid balance may only play a role in people with much higher cytokine concentrations, as can be found in acute inflammatory and infectious diseases. They have not been investigated in HCs or in patients with AN yet. In this context, it is of note that the cited articles refer to experimental and clinical studies from gastrointestinal, renal, lung, and skin research and thus may not be relevant to the physiological regulation of water balance in healthy people and people with eating disorders.

Nevertheless, given that patients with AN had decreased ICW, ECW, and TBW in this and previous studies, and given that some cytokines were statistically associated with parameters of fluid balance in the whole sample, it seems justified to have a more detailed look at the associations between cytokines and ICW, ECW, and TBW in patients with AN. In AN participants, the most notable result is the statistically significant and negative correlation of IL-10 with ICW, ECW, and TBW. IL-10 also correlated negatively with body weight. Thus, IL-10 could be indirectly connected with ICW, ECW, and TBW via the low body weight.

The association of IL-10 with water volumes can be explained in several ways: (a) IL-10 may influence water balance by decreasing fluid intake or by increasing water excretion, (b) hypohydration may lead to an increase of IL-10 production, or (c) weight could influence both IL-10 levels and water balance. However, as body water contributes to weight, these two parameters are not independent. IL-10 regulates the growth and differentiation of B cells, NK cells, cytotoxic and helper T cells, mast cells, granulocytes, dendritic cells, keratinocytes, and endothelial cells [33]. It has also been proposed that it reduces thirst: Acacia catechu, a traditional thirst quencher of South India, has been reported to increase IL-10 production [34], but also a direct effect of IL-10 on thirst was seen in a previous study [35]. Therefore, it is unclear whether IL-10 can reduce thirst in humans. IL-10 is highly secreted in mucosal tissues such as the gut, and studies have confirmed the role of IL-10 in controlling gut inflammation and establishing mutually beneficial commensalism of intestinal microbiota with the mammalian hosts [36]. IL-10 also affects kidney health and function. It can be protective under some conditions but has also been reported to aggravate defects in renal function [37]. Overall, however, it is unclear whether IL-10 can lead to an increased fluid loss via the gut or the kidneys. An additional factor that may influence both IL-10 levels and water content of the body is physical exercise. IL-10 production has been reported to increase after physical exercise [38–40], and physical exercise is also well known as a cause of hypohydration [41]. However, IL-10 levels did not differ between patients with AN and HCs in this study [23]. Therefore, this assumption would be speculation. In fact, we can assume that about 30 percent of people with AN do not exercise regularly [42]. Taken together, there is no literature on the influence of IL-10 on the bodily water content or vice versa.

We found that IL-6 and IL-15 levels were significantly higher in patients with AN compared to HCs, whereas TNF-*β* concentrations were lower in people with AN. The finding of elevated IL-6 levels is in line with previous meta-analytic research [10]. Interestingly, people suffering from obesity have also been reported to have an increased production of proinflammatory cytokines such as IL-6. In people with obesity, these proinflammatory cytokines are most likely released by inflammatory cells infiltrating the adipose tissue [43]. This hypothesis that an increase in proinflammatory cytokines is a consequence of inflammatory cells in the adipose tissue cannot be applied to people with AN. In these patients, it has been speculated that increased oxidative stress, chronic physiological and psychological stress, changes in the intestinal microbiota, and an abnormal bone marrow microenvironment contribute to changes in their immune system and thus in cytokine production [44].

Our study has some limitations. Our sample size is relatively small, and we did not control for multiple testing, i.e., our study is exploratory. Our cross-sectional approach limits speculation on the direction of causality. Measurement of body water by BIA uses electrical impedance, i.e., it does not directly determine body water [45], and thus, dehydration and electrolyte imbalances may lead to problems with measurement accuracy [46]. However, it has been suggested that these issues are more related to the measurement of fat mass than to the measurement of body water [46].

## 5. Conclusion

In conclusion, our results suggest an interaction between body water distribution and the production of certain cytokines, namely, GM-CSF, MCP-4, IL-4, IFN-*γ*, IL-6, and IL-10. The changes of body water and its distribution may occur via the gut, kidneys, or skin due to AN-associated inflammatory processes. They may be due to the physical health consequences of AN or be in part associated with overexercising in these patients. Further research that employs a larger sample size and a longitudinal approach to address the direction of causality is needed.

## Figures and Tables

**Table 1:** Demographic, anthropometric, and clinical characteristics for AN participants and HCs.

		Healthy controls		Anorexia nervosa	Cross-sectional comparison
	*N*	Mean ± SD	*N*	Mean ± SD	
Age (years)	11	24.82 ± 3.52	27	31.48 ± 11.40	*U* = 144, *z* = −1.36, *p* = 0.1735
EDE-Q global	11	0.73 ± 0.74	27	4.20 ± 1.27	*U* = 85.5, *z* = −4.87, *p* < 0.0010
BMI (kg/m^2^)	11	21.13 ± 1.72	27	15.33 ± 2.25	*t* (38) = 7.88, *p* < 0.0010
Body fat (%)	10	17.83 ± 6.29	27	7.76 ± 6.07	*U* = −22, *z* = 3.63, *p* = 0.0003
ICW (l)	11	22.14 ± 2.39	27	17.47 ± 3.39	*t* (36) = 4.15, *p* = 0.0002
ECW (l)	11	13.15 ± 1.45	27	10.64 ± 1.80	*t* (36) = 4.11, *p* = 0.0002
TBW (l)	11	35.28 ± 3.82	27	28.11 ± 5.13	*t* (36) = 4.18, *p* = 0.0002
ECW/TBW ratio	11	0.37 ± 0.01	27	0.38 ± 0.01	*t* (36) = −1.66, *p* = 0.1050

Abbreviations: SD = standard deviation; EDE-Q = Eating Disorder Examination-Questionnaire; BMI = body mass index; ICW = intracellular water; ECW = extracellular water; TBW = total body water.

**Table 2:** Number of participants with undetectable cytokines for each cytokine in the whole sample (*n* = 38) with median serum concentrations (pg/ml, with interquartile range) of cytokines for HC (*n* = 11) and AN (*n* = 27) participants, with a significance value of group comparison.

	Undetectable^#^ (*n* (%))	Healthy controls		Anorexia nervosa		*p*
		*N*	Median (IQR^†^)	*N*	Median (IQR^†^)	
Eotaxin	0	11	208.55 (160.77, 252.76)	27	175.47 (155.93, 279.31)	0.7113
Eotaxin-3	0	11	21.36 (17.35, 29.27)	27	15.15 (11.36, 25.58)	0.1147
GM-CSF	5 (13.2%)	11	0.24 (0.06, 0.52)	22	0.19 (0.12, 0.29)	0.6060
IFN-*γ*	0	11	3.28 (2.68, 4.60)	27	4.64 (2.89, 8.61)	0.1185
IL-1*α*	0	11	1.13 (0.65, 2.09)	27	1.01 (0.65, 1.29)	0.3761
IL-1*β*	10 (26.3%)	7	0.21 (0.04, 0.39)	21	0.19 (0.07, 0.25)	0.6907
IL-2	19 (50%)	6	0.12 (0.04, 0.28)	13	0.15 (0.09, 0.25)	0.9301
IL-4	2 (5.3%)	11	0.07 (0.04, 0.13)	25	0.05 (0.03, 0.08)	0.2717
IL-5	4 (10.5%)	10	1.12 (0.92, 1.74)	24	1.29 (0.72, 1.72)	0.7913
IL-6	0	11	0.30 (0.13, 0.42)	27	0.49 (0.35, 1.25)	0.0054
IL-7	0	11	15.45 (10.18, 17.44)	27	12.58 (10.46, 17.27)	0.9359
IL-8	0	11	37.24 (14.00, 118.30)	27	23.09 (10.27, 103.21)	0.3106
IL-10	0	11	0.24 (0.08, 0.26)	27	0.28 (0.11, 0.37)	0.3422
IL-12/IL-23p40	0	11	117.69 (81.15, 138.62)	27	92.00 (68.06, 118.65)	0.1185
IL-12p70	4 (10.5%)	9	0.15 (0.12, 0.19)	25	0.19 (0.11, 0.36)	0.5068
IL-13	14 (36.8%)	7	3.26 (2.13, 8.50)	17	2.44 (1.17, 4.30)	0.1197
IL-15	0	11	2.44 (2.23, 2.57)	27	2.90 (2.70, 3.51)	0.0021
IL-16	0	11	178.76 (138.29, 218.50)	27	183.59 (145.14, 339.36)	0.2274
IL-17A	0	11	1.78 (1.26, 3.20)	27	1.91 (1.08, 2.59)	0.9743
IP-10	0	11	110.93 (81.79, 151.33)	27	115.99 (98.45, 207.33)	0.3937
MIP-1*α*	0	11	27.16 (21.11, 33.29)	27	23.02 (20.10, 35.20)	0.9230
MIP-1*β*	0	11	115.67 (62.12, 141.36)	27	81.06 (65.78, 110.10)	0.2809
MCP-1	0	11	208.55 (165.87, 292.65)	27	191.53 (164.41, 241.73)	0.3937
MCP-4	0	11	149.14 (93.31, 195.49)	27	120.65 (86.10, 169.37)	0.3937
TARC	0	11	477.35 (221.30, 607.12)	27	370.33 (263.01, 641.46)	0.9359
TNF-*α*	0	11	1.59 (1.16, 2.51)	27	1.64 (1.34, 2.42)	0.8343
TNF-*β*	0	11	0.86 (0.70, 1.21)	27	0.60 (0.49, 0.69)	0.0096

^#^Below fit curve range. ^†^25th and 75th percentile reported. Abbreviations: IQR = interquartile range; GMCSF = granulocyte-macrophage colony-stimulating factor; IFN-*γ* = interferon-*γ*; IL = interleukin; IP-10 = interferon *γ*-induced protein-10; MIP = macrophage inflammatory protein; MCP = monocyte chemoattractant protein; TARC = thymus and activation-regulated chemokine; TNF = tumor necrosis factor (TARC).

**Table 3:** Correlations (*r*_s_) between cytokine concentrations and water distribution variables in the whole sample.

	ICW	ECW	TBW	ECW/TBW ratio
Eotaxin	0.03	0.08	0.06	-0.04
Eotaxin-3	0.17	0.14	0.18	-0.34^∗^
GM-CSF	0.43^∗^	0.45^∗^	0.43^∗^	-0.13
IFN-*γ*	-0.34^∗^	-0.35^∗^	-0.36^∗^	0.25
IL-1*α*	0.33^∗^	0.31	0.32^∗^	-0.25
IL-1*β*	0.06	0.10	0.04	0.34
IL-2	0.09	0.04	0.11	0.29
IL-4	0.38^∗^	0.43^∗^	0.40^∗^	-0.01
IL-5	-0.17	-0.09	-0.11	0.30
IL-6	-0.36^∗^	-0.34^∗^	-0.37^∗^	0.32
IL-7	-0.02	-0.05	-0.01	-0.14
IL-8	-0.05	-0.04	-0.05	0.02
IL-10	-0.45^∗^	-0.45^∗^	-0.46^∗^	0.31
IL-12/IL-23p40	0.18	0.12	0.16	-0.32^∗^
IL-12p70	-0.02	-0.05	-0.02	-0.02
IL-13	0.17	0.07	0.12	-0.22
IL-15	-0.33^∗^	-0.30	-0.32	0.23
IL-16	-0.13	-0.13	-0.14	0.04
IL-17A	0.04	-0.04	0.01	-0.29
IP-10	-0.11	-0.16	-0.13	-0.13
MIP-1*α*	0.15	0.05	0.10	-0.34^∗^
MIP-1*β*	0.22	0.17	0.20	-0.22
MCP-1	0.09	0.08	0.11	-0.18
MCP-4	0.33^∗^	0.38^∗^	0.37^∗^	-0.22
TARC	-0.14	-0.12	-0.11	-0.06
TNF-*α*	0.03	0.02	0.04	-0.09
TNF-*β*	0.33^∗^	0.26	0.32	-0.30

∗ indicates *p* < 0.05. Abbreviations: GMCSF = granulocyte-macrophage colony-stimulating factor; IFN-*γ* = interferon-*γ*; IL = interleukin; IP-10 = interferon *γ*-induced protein-10; MIP = macrophage inflammatory protein; MCP = monocyte chemoattractant protein; TARC = thymus and activation-regulated chemokine; TNF = tumor necrosis factor (TARC); ICW = intracellular water; ECW = extracellular water; TBW = total body water.

**Table 4:** Correlations (*r*_s_) between cytokine concentrations and water distribution variables in the AN sample only.

	ICW	ECW	TBW	ECW/TBW ratio
Eotaxin	0.02	0.13	0.09	0.04
Eotaxin-3	-0.11	-0.14	-0.10	-0.28
GM-CSF	0.38	0.39	0.36	-0.13
IFN-*γ*	-0.20	-0.21	-0.22	0.31
IL-1*α*	0.26	0.20	0.22	-0.28
IL-1*β*	-0.05	-0.05	-0.11	0.40
IL-2	-0.01	-0.04	0.02	0.51
IL-4	0.36	0.44^∗^	0.39	0.07
IL-5	-0.38	-0.28	-0.31	0.39
IL-6	-0.23	-0.23	-0.26	0.25
IL-7	0.01	-0.03	0.02	-0.17
IL-8	-0.26	-0.28	-0.28	0.09
IL-10	-0.42^∗^	-0.48^∗^	-0.44^∗^	0.25
IL-12/IL-23p40	0.00	-0.10	-0.04	-0.34
IL-12p70	0.12	0.05	0.10	-0.08
IL-13	0.10	-0.06	0.01	-0.09
IL-15	-0.20	-0.11	-0.15	0.25
IL-16	0.03	-0.00	-0.00	-0.04
IL-17A	0.18	0.04	0.13	-0.37
IP-10	0.06	-0.02	0.03	-0.19
MIP-1*α*	0.19	0.04	0.14	-0.43^∗^
MIP-1*β*	-0.01	-0.09	-0.06	-0.26
MCP-1	-0.09	-0.10	-0.06	-0.11
MCP-4	0.30	0.44^∗^	0.36	-0.02
TARC	-0.14	-0.12	-0.13	-0.03
TNF-*α*	-0.02	-0.05	-0.01	-0.16
TNF-*β*	0.12	0.04	0.11	-0.19

∗ indicates *p* < 0.05. Abbreviations: GMCSF = granulocyte-macrophage colony-stimulating factor; IFN-*γ* = interferon-*γ*; IL = interleukin; IP-10 = interferon *γ*-induced protein-10; MIP = macrophage inflammatory protein; MCP = monocyte chemoattractant protein; TARC = thymus and activation-regulated chemokine; TNF = tumor necrosis factor (TARC); ICW = intracellular water; ECW = extracellular water; TBW = total body water.

## Data Availability

The ethics approval of this study does not include sharing the underlying data set. However, Iain Campbell (email: iain.campbell@kcl.ac.uk) may be contacted for specific requests.
